# Life Cycle and Phylogeography of True Truffles

**DOI:** 10.3390/genes13010145

**Published:** 2022-01-14

**Authors:** Jiao Qin, Bang Feng

**Affiliations:** 1 Key Laboratory of Economic Plants and Biotechnology, Kunming Institute of Botany, Chinese Academy of Sciences, Kunming 650201, China; qinjiao@mail.kib.ac.cn; 2 CAS Key Laboratory for Plant Diversity and Biogeography of East Asia, Kunming Institute of Botany, Chinese Academy of Sciences, Kunming 650201, China; 3 Yunnan Key Laboratory for Fungal Diversity and Green Development, Kunming 650201, China

**Keywords:** life cycle, mating type, genetic structure, glaciation, river isolation, ascocarp-associated bacteria

## Abstract

True truffle (*Tuber* spp.) is one group of ascomycetes with great economic importance. During the last 30 years, numerous fine-scale population genetics studies were conducted on different truffle species, aiming to answer several key questions regarding their life cycles; these questions are important for their cultivation. It is now evident that truffles are heterothallic, but with a prevalent haploid lifestyle. Strains forming ectomycorrhizas and germinating ascospores act as maternal and paternal partners respectively. At the same time, a number of large-scale studies were carried out, highlighting the influences of the last glaciation and river isolations on the genetic structure of truffles. A retreat to southern refugia during glaciation, and a northward expansion post glaciation, were revealed in all studied European truffles. The Mediterranean Sea, acting as a barrier, has led to the existence of several refugia in different peninsulas for a single species. Similarly, large rivers in southwestern China act as physical barriers to gene flow for truffles in this region. Further studies can pay special attention to population genetics of species with a wide distribution range, such as *T. himalayense*, and the correlation between truffle genetic structure and the community composition of truffle-associated bacteria.

## 1. Introduction

True truffles refer to hypogenous ascocarps produced by species of the genus *Tuber*. This genus consists of 11 major phylogenetic clades [1] and approximately 200 species [2], and plays an important ecological role as an ectomycorrhizal partner of several plant families, such as Fagaceae and Pinaceae [2]. Many true truffles from Europe, such as *T. melanosporum* (the Périgord black truffle), *T. magnatum* (the Italian white truffle), *T. aestivum* (the Burgundy truffle) and *T. borchii* (the bianchetto truffle), are highly prized edible species known for their aromatic and gustatory qualities. The Chinese black truffles, composed of *T. indicum* and *T. himalayense*, are widely collected, consumed, and exported to Europe [3]. Driven by their economic importance, many truffles, especially *T. melanosporum*, were successfully cultivated, not only in their country of origin, but in non-native countries, such as Australia, New Zealand, the United States and South Africa [4]. In this process, many questions have arisen regarding the life cycle of true truffles, some of which have been answered by population genetics and genomic studies on representative species, while others remain unresolved [5]. In contrast, many truffle species have a narrow distribution range. Moreover, truffles produce below-ground ascocarps and depend on truffle-hunting animals for the dispersal of spores. This raises the question of how the distribution pattern of true truffles affects their genetic structure. Many have attempted to answer this question using the powerful tool of population genetics.

In this review, we summarized more than three decades of population genetics research on true truffles, highlighting the following two issues: (1) the life cycle of true truffles, and (2) the genetic structure of true truffles, and its association with historical climatic and tectonic variations.

## 2. A Brief History of Population Genetics Studies on True Truffles

The origin of population genetics studies on true truffles can be traced back to the late 1980s. In 1989, Pacioni and Pomponi found great genetic stability in *T. melanosporum* and *T. brumale* by using allozyme electrophoresis; however, significant genetic heterogeneity was detected in *T. aestivum* and *T. mesentericum* [6]. Later, they analyzed the genetic patterns of *T. aestivum* and *T. mesentericum* in Italian populations using multi-locus electrophoresis, revealing that the two species can be delimited using molecular evidence [7]. Following this study, population genetics studies have been carried out in several truffle species, utilizing different types of gene makers. In brief, the genetic markers used include random amplified polymorphic DNA (RAPD) [8], simple sequence repeat (SSR, microsatellite) [9,10,11,12,13,14,15,16], amplified fragment length polymorphism (AFLP) [10], inter-simple sequence repeat (ISSR) [17], mating type loci [11,12,13,14,15] and single-nucleotide polymorphisms (SNPs) from different DNA fragments, such as internal transcribed spacers (ITS) [18,19,20,21], beta-tubulin gene [20] and SNPs at the genomic scale [22]. The major concerns of these studies are (1) species delimitation of several species complex, e.g., *T. indicum* [3,20,21], *T. aestivum* [23], *T. borchii* [24] and *T. brumale* [25], (2) life cycle of true truffles [9,10,13,14,15], and (3) genetic structure of several truffle species [16,17,18,19,25,26,27].

## 3. Life Cycle of True Truffles

### 3.1. True Truffles Are Heterothallic

The first question that must be answered regarding the life cycle of a fungus is its reproductive system. Generally, there are two paradigmatic sexual systems in fungi: heterothallism and homothallism [28,29]. It is expected that heterozygosity will be detected in heterothallic species while using codominant genetic markers (such as RAPD, AFLP and SSR), as such species contain both maternal and paternal genotypes. Until genomic information is available for truffle species, this is the theoretical basis for inferring the reproductive system of true truffles through population genetics studies. However, until 2005, both *T. melanosporum* and *T. magnatum* were considered to be self-compatible, or highly inbred species, as no heterozygous ascocarp was detected when genotyped using codominant makers [8,19,26,30]. Nonetheless, extensive genetic exchange was detected in *T. magnatum* populations [31], leading to the hypothesis that the majority of the ascocarps of *T. magnatum*, the so-called gleba, are composed of haploid maternal material, while ascospores, which have cell walls that are difficult to disrupt during DNA extraction, contain both paternal and maternal information. This hypothesis was supported by SSR genotyping on gleba and asci (including ascospores) separately, clearly indicating that *T. magnatum* is an outcross species [9]; later, the same phenomenon was reported for *T. melanosporum* [10]. In 2010, genome sequencing provided direct evidence that *T. melanosporum*, bearing only one mating type idiomorph in a single ascocarp, is heterothallic, and thus an obligate outcrossing species [32]. This was further confirmed by screening mating-type idiomorphs in different ascocarps of *T. melanosporum* [33]. Other species were later shown to be heterothallic based on genome sequences (*T. magnatum* and *T. aestivum*) [34], or sequences of mating type genes (*T. indicum* species complex [35] and *T. borchii* [36]).

### 3.2. Ectomycorrhizal Mycelia and Germlings from Ascospores Contribute as Maternal and Paternal Partners, Respectively

Mating-type genes were used in population genetics studies of true truffles (particularly *T. melanosporum*) soon after they were reported, together with SSR genotyping, a combination that has greatly improved our understanding of the life cycle of true truffles. By simultaneously genotyping ectomycorrhizas (ECMs) and ascocarps in fine-scale areas, it has been shown that gleba of ascocarps and surrounding ECMs are both formed by haploid mycelia [11,12], and that they have the same mating type profile (either MAT1-1 or MAT1-2), and the same SSR genotype, whereas ascospores show several genotypes that have never been detected in ECMs and gleba. This suggests that gleba, the maternal material of truffles, preferentially comes from strains that have colonized the roots of their hosts. This also raises the question of where the parental partner comes from [12]. There are some potential origins, including but not limited to: (1) mycelia from germinated ascocarps or nearby ECMs [14,15], (2) free-living mycelia in soil [15], and (3) mycelia that exist as endophytes in non-host plants [5]; *Tuber melanosporum* and *T. aestivum* can endophytically colonize roots of non-ectomycorrhizal plants [13,37,38]. However, it was later shown that endophytisms in both *T. melanosporum* and *T. aestivum* are of a maternal feature [13]. Instead, some studies showed that germinated ascocarps and free-living mycelia may serve as paternal material. By genotyping ascocarps consecutively over a four-year period (2010–2014), it was found that paternal genotypes, unlike maternal ones, cannot persist over the years, and cannot be detected from ECMs [14]. Therefore, Taschen et al. [14] speculated that germlings from the soil spore bank act as paternal partners, based on their own observations, and that mating type idiomorph supplemental to surrounding ECMs can rarely be detected in soil [12]. Contrasting with this study, De la Varga et al. [15] detected signals of the persistence of paternal genotypes over years, and the existence of both mating type idiomorphs in soil patches, suggesting that both germinating ascospores and soil mycelia can act as paternal genotypes; nonetheless, both studies supported that germinating ascospores may play an important role as a paternal genotype of *T. melanosporum*. A later study on *T. borchii* led to the same hypothesis [39]. Interestingly, it was found that up to 42% of fruitbodies remain unremoved in managed truffle grounds [40], indicating that there is enough spore bank in soil to act as paternal partners. Unfortunately, this still lacks direct evidence from genotyping soil DNAs, using both mating type and SSR markers. To fulfill this gap, two issues need to be addressed, (1) genotyping should be carried out in different time scales, as the time for the joining of paternal gametes is still questionable [5], and (2) technical improvements to obtain DNAs from ascospores in soil, or to separate ascospores from soil, are needed.

**Figure 1:**
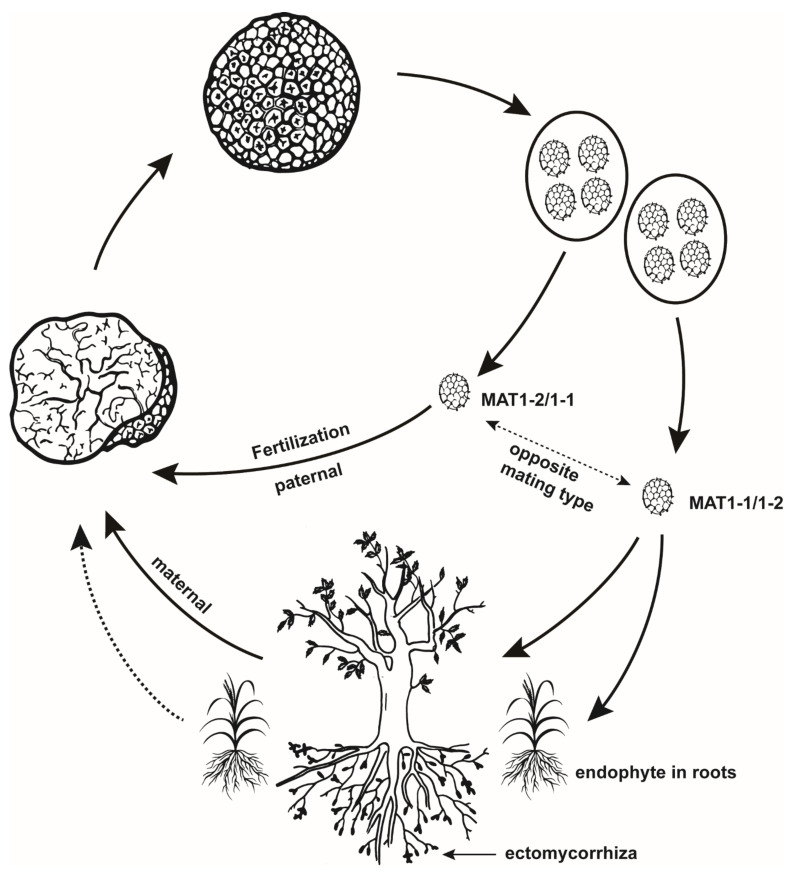
A scheme showing the life cycle of true truffles.

Here, we present a scheme for the life cycle of truffles (Figure 1), based on the above evidence. We must note that endophytism in the non-host plant likely makes no direct contribution to the formation of ascocarps; instead, it can improve the production of truffles by reducing the germination and growth of weeds [41].

### 3.3. Strains with Opposite Mating Type Idiomorphs Compete for Existence on Host Roots

In exploring the distribution pattern of mating types in natural *T. melanosporum* plantations, Rubini et al. [12] found identical mating types and SSR profiles under a single host plant. Furthermore, it was shown that “the minimum distance between sites where ECM of different mating types were detected was 50 m”, indicating a clear spatial segregation of mating types [12]. The same authors also screened the distribution patterns of mating-type idiomorphs in nursery-grown plants. The results showed that, at the beginning of inoculation with *T. melanosporum* ascocarps (six months), nearly all seedlings can form ECMs with strains featuring both mating type idiomorphs, but in nearly half of these seedlings, only one idiomorph could be detected 18 months after inoculation [12]. Similarly, a study from Australia [42] showed that the two idiomorphs were detectable in all two-year-old seedlings, while only one idiomorph was detectable in nearly half of the trees from plantations established four to ten years ago. Murat et al. [11] reported that all mycorrhizas displayed the same mating type in large soil patches of up to 15 m^2^, by investigating two old plantations of *T. melanosporum,* set up for over 15 years. These observations suggest that strains with opposite mating types compete for existence in host roots in different timelines, and result in the spatial separation of mating types. Similarly, the segregation of mating types was observed in *T. aestivum*, although the tendency is less pronounced than in *T. melanosporum* [43]. Although several hypotheses have been proposed [5,44], the reasons or mechanisms for the competition, and its potential contribution in the reproduction of truffles, remain unclear.

## 4. Genetic Structure of True Truffles

### 4.1. The Last Glaciation Has a Common Influence on the Genetic Structure of European Truffles

In addition to fine-scale population genetics studies aimed at revealing the reproductive pattern of true truffles, a number of large-scale studies have been conducted to explore their genetic diversity and genetic structure. Many European species have attracted the attention of researchers, and most studies have addressed how the last glaciation has affected the genetic structure of these truffles. Although the distribution ranges of the studied truffle species differed, and different molecular markers were used in these studies, it was shown that almost all species studied experienced a bottleneck during the glacial period, when they retreated into southernmost refugia, and a post-glacial expansion to northern areas [10,16,17,18,19,25,27,31,45].

Among these, *T. melanosporum*, *T. magnatum*, *T. brumale* and *T. mesentericum* are four species with relatively narrow distribution ranges. In *T. melanosporum*, analyses of Italian and French samples, using either SNPs from ITS or SSR genotyping, identified higher levels of genetic diversity in southernmost populations, supporting the Italian Peninsula as its refugium during the last glaciation [10,19]. For samples from Spain, high levels of genetic diversity could be detected in southern, central, and northern populations using ISSR genotyping, suggesting that the Iberian Peninsula may represent another refugium of this species [17]. Unfortunately, different genetic markers were used, and so those results cannot be combined to reveal the genetic structure of *T. melanosporum,* covering its full distribution range. Similarly, SSR genotyping suggested that the Italian [16,31] and the Balkan Peninsulas [16] act as two refugia of *T. magnatum*. *Tuber brumale*, with a similar distribution pattern to *T. magnatum*, was proven to be a species complex containing two species, *T. brumale* and *T. cryptobrumale*, the latter mainly distributed in the Carpathian Basin [25,45]. A study based on SNPs from ITS sequences showed that *T. brumale* harbored two main haplotype groups, suggesting the existence of two refugia for this species, Eastern Europe refugium (the Balkan Peninsula) and Western Europe refugium, whereas *T. cryptobrumale* may have survived in the Carpathian refugium during the last glaciation [25]. Similarly, *T. mesentericum* can be divided into three main clades [46,47] representing three cryptic species: *T. mesentericum*, *T. bituminatum* and *T. suave* [47]. The phylogeographic structure of the *T. mesentericum* species complex was not discussed in depth in the available studies [46,47]. Nevertheless, the narrow distribution of *T. bituminatum* and *T. suave* in southern Europe (mainly Italy) suggests that the Italian peninsula has played an important role in the speciation and maintenance of these two cryptic species. Unlike the three aforementioned species, *T. aestivum* can be found in Europe [27] and Turkey [48]. SSR genotyping on European samples identified four genetic groups without significant geographical isolation, indicating the existence of ecotypes [27]. In contrast, analyses on ITS sequences showed a higher level of genetic diversity in Turkish and southern European populations than in northern European populations, suggesting that Turkey and southern Europe may have acted as refugia for *T. aestivum* [18].

**Figure 2:**
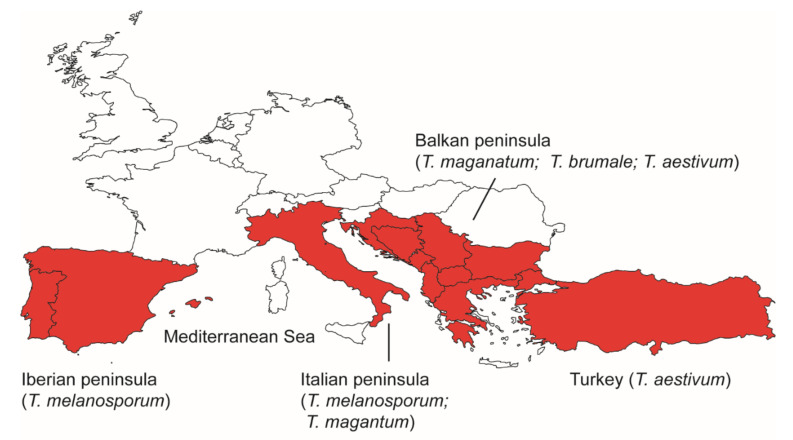
A map showing refugia for different European truffle species during the last glaciation.

Taking all these observations together, it is evident that southern Europe, including the Iberian, Italian and Balkan Peninsulas, and Turkey have acted as refugia for different truffle species during the last glacial period (Figure 2). The existence of multiple refugia isolated by the Mediterranean Sea for a single species [16,18] indicates that the Mediterranean Sea may act as a physical barrier to gene flow for truffle species. However, there is evidence of gene flow between different peninsulas for *T. magnatum* [16], or between Europe and Turkey in *T. aestivum* [18]. It was hypothesized that such migrations were introduced by land bridges [16] or by human activities [18].

### 4.2. Drainage Isolation Contributes to Genetic Structure Formation of Chinese Black Truffle

The Chinese black truffle, distributed mainly in East Asia, is one species complex that received broad interest in population genetics studies. This species complex was separated into two phylogenetic clades that represent two species: *T. indicum* and *T. himalayense* [49]. However, these two clades were also treated as two ecotypes of a single species; *T. indicum*, as “a west–east sinuous line between the two groups”, has been observed [20]. It was later shown that both clades have a sympatric distribution in several localities [3], but harbor “sequence variations and rearrangements in both coding and non-coding regions” in their mating type idiomorphs [35], suggesting that they should be two species with significant reproductive isolations [3]. This treatment was further supported by SSR genotyping on population samples from southwestern China [21], and phylogenetic analyses on samples from China and Japan by using mating-type loci and other DNA fragments [50].

**Figure 3:**
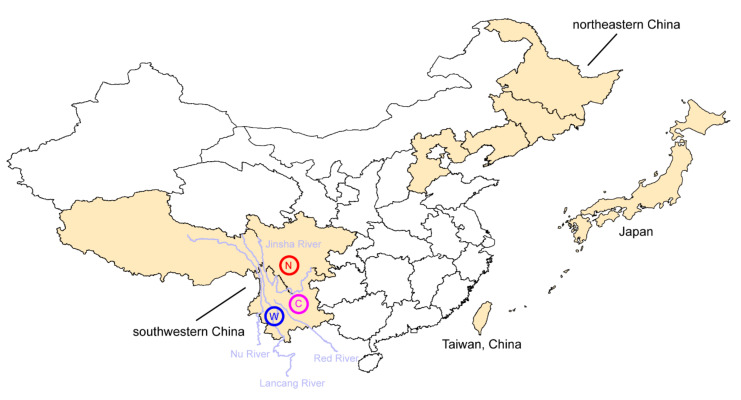
A map showing the distribution range (in yellow) and biogeographic patterns of Chinese black truffles. *Tuber indicum* can be found in southwestern China and three haplotype groups (N, north; C, central and W, west) were revealed, while *T. himalayense* can be found in southwestern China, northwestern China, Taiwan island of China and Japan.

In southwestern China, *T. indicum* and *T. himalayense* are mainly distributed in the “Three Parallel Rivers” region. Population genetics analyses using SNPs from four DNA fragments revealed three major haplotype groups, namely Group W (west), C (central) and N (north), isolated by the paleo-Red River and modern Jinsha River (the upper region of Yangtze River), in *T. indicum* (Figure 3). This indicates that both paleo and modern drainage systems have contributed to the formation of its genetic structure by acting as physical barriers to gene flows between populations. In contrast, no significant geographic structure was detected in *T. himalayense*, likely due to the lower evolutionary rates of this species, at least in the DNA fragments used in the population genetics study [3]. In fact, a significant “isolation by rivers” genetic structure could be detected in *T. himalayense* while using SNPs from the whole genome to infer the phylogeographical structure of this species (author’s unpublished data). Therefore, drainage isolations have served as a key driving factor for the formation of genetic structure in both *T. indicum* and *T. himalayense*.

Notably, *T. himalayense* is distributed over a wide range in East Asia, including southwestern China, northeastern China [51], Taiwan Island of China (the so-called *T. formosanum*) and Japan [50] (Figure 3). This discontinuous distribution pattern makes for an ideal system for studying the evolution of truffles over a large geographic range. However, studies on this species have only been conducted in southwestern China [3,21] and Japan [52]. Further studies covering its entire distribution range would help us to better understand its evolutionary history.

## 5. Conclusions and Perspectives

After more than 30 years of research on the population genetics of true truffles, our understanding of the life cycle and genetic structure of truffle species has been improved to a great extent. It is now clear that truffle species are heterothallic but with a prevalent haploid lifestyle, similar to those of true morels (*Morchella* spp.) [29]. Strains form ectomycorrhizas contribute as the maternal partner, while germinating ascospores act as the paternal partner, although additional direct evidence is still needed to support this view. Concerning their genetic structure, most European truffle species retreated into the southern refugia for survival during the last glacial period, and expanded northward after glaciation. The isolation of the Mediterranean Sea could have led to the existence of several refugia in different peninsulas for a single truffle species. Similarly, large rivers in southwestern China act as physical barriers to gene flows for *T. indicum* and *T. himalayense*, thus contributing a lot to the genetic structure of these two species. However, how the discontinuous distribution of *T. himalayense* in China (including mainland China and Taiwan) and Japan can influence the genetic structure of this species is still an open question. This can be answered by conducting population genomics on samples gathered from different regions.

Abundant and diverse ascocarp-associated bacteria were reported from several species of true truffles, such as *T. melanosporum* [53,54,55], *T. maganatum* [56,57,58], *T. aestivum* [59,60], *T. borchii* [61], *T. indicum* species complex [51] and *T. pseudohymalayense* [62]. Remarkably, studies have shown that the genus *Bradyrhizobium* is dominant in most studied true truffle species [51,63], but can be rarely found in false truffes [64], and that a single *Bradyrhizobium* species is able to form symbiotic reactions with truffles from different continents [51]. This raises a previously unexplored question: what if truffles and their associated *Bradyrhizobium* species have co-evolved at the population level? Furthermore, ascocarp-associated bacteria have been shown to play a role in aroma formation [65,66] and nitrogen fixation [51,56]. Intriguingly, it has been shown that the genetic diversity of truffles can influence the variation in their aroma [67,68], and that truffle species can select their associated bacterial communities [62,63]. It will be interesting to explore how the community composition of ascocarp-associated bacteria, the genetic structure of truffles, and aroma variability are linked. Fortunately, it has come to our attention that the journal *Diversity* is preparing a Special Issue on “Genetic diversity of truffle species and soil microbial interactions”. Hopefully, research work on this issue will bring us answers to the above questions.

## Data Availability

Not applicable.
